# Coupling between Polymer Conformations and Dynamics Near Amorphous Silica Surfaces: A Direct Insight from Atomistic Simulations

**DOI:** 10.3390/nano11082075

**Published:** 2021-08-16

**Authors:** Petra Bačová, Wei Li, Alireza F. Behbahani, Craig Burkhart, Patrycja Polińska, Manolis Doxastakis, Vagelis Harmandaris

**Affiliations:** 1 Institute of Applied and Computational Mathematics (IACM), Foundation for Research and Technology Hellas (FORTH), GR-70013 Heraklion, Greece; af.behbahani@iacm.forth.gr; 2 Computation-Based Science and Technology Research Center, The Cyprus Institute, 20 Constantinou Kavafi Str., Nicosia 2121, Cyprus; 3 Department of Chemical and Biomolecular Engineering, University of Tennessee, Knoxville, TN 37996, USA; wli52@utk.edu (W.L.); edoxasta@utk.edu (M.D.); 4 The Goodyear Tire and Rubber Company, Akron, OH 44305, USA; craig.burkhart@goodyear.com; 5 Goodyear S.A., L-7750 Colmar-Berg, Luxembourg; patrycja_polinska@goodyear.com; 6 Department of Mathematics and Applied Mathematics, University of Crete, GR-70013 Heraklion, Greece

**Keywords:** atomistic simulations, interphase, polymer dynamics, films, rough silica interfaces

## Abstract

The dynamics of polymer chains in the polymer/solid interphase region have been a point of debate in recent years. Its understanding is the first step towards the description and the prediction of the properties of a wide family of commercially used polymeric-based nanostructured materials. Here, we present a detailed investigation of the conformational and dynamical features of unentangled and mildly entangled *cis*-1,4-polybutadiene melts in the vicinity of amorphous silica surface via atomistic simulations. Accounting for the roughness of the surface, we analyze the properties of the polymer chains as a function of their distance from the silica slab, their conformations and the chain molecular weight. Unlike the case of perfectly flat and homogeneous surfaces, the monomeric translational motion parallel to the surface was affected by the presence of the silica slab up to distances comparable with the extension of the density fluctuations. In addition, the intramolecular dynamical heterogeneities in adsorbed chains were revealed by linking the conformations and the structure of the adsorbed chains with their dynamical properties. Strong dynamical heterogeneities within the adsorbed layer are found, with the chains possessing longer sequences of adsorbed segments (“trains”) exhibiting slower dynamics than the adsorbed chains with short ones. Our results suggest that, apart from the density-dynamics correlation, the configurational entropy plays an important role in the dynamical response of the polymers confined between the silica slabs.

## 1. Introduction

Hybrid materials containing polymer–solid interfaces have attracted considerable attention in the last few decades due to the large variety of their possible adaptations and a wide span of applications. Polymer nanocomposites and polymer films are among the most populated types of hybrid polymer materials used for instance, in the tire industry as coatings or in membrane and separation technologies [1,2]. The versatility of these materials comes from the fact that their properties can be tuned by using a broad set of parameters, such as polymer type and its molecular weight; characteristics of the solid interface (e.g., its roughness or shape and mutual interaction with the polymer); dispersion state; and concentration of nanofillers in the case of nanocomposites or film thickness in the case of polymer films. Quantitative similarities have been found between both types of hybrid materials [3,4] owing mostly to the similar polymer behavior in the vicinity of the surfaces. In general, in the case of favorable polymer–solid interactions, the polymer chains adsorb on the surface, forming conformations commonly known as trains, loops and tails. Trains are formed by consecutive adsorbed segments on the surface, the loops consist of segments connecting the trains and the tails are the end sections of adsorbed chains. Consequently, the chain dynamics are coupled with these conformational states, resulting in the formation of heterogeneous dynamical regions in the material [5,6,7]. This generally valid idea is a good starting point in probing the structure–dynamics relation in polymer nanocomposites and films. However, due to the large number of the parameters affecting the properties and the experimental challenges related to the sample preparation [8,9,10], which in part stem from the irregularity of the surface [11,12,13,14], many essential theoretical questions remain to be addressed [15,16].

Molecular simulation techniques proved to be a very powerful tool in the pursuit of a detailed description of the interfacial phenomena for nanostructured materials, as they allow for a full control of the preparation process and for a spatio-temporal analysis of the polymer conformational and dynamical behavior. Generic bead-spring and Monte Carlo models, which do not embody any specific chemical characteristics of the material, have been implemented for examining universal trends in properties of hybrid materials. These models are able to capture basic polymer features such as polymer flexibility [17,18] or conformational changes at the interface [19,20], and by setting up an explicit type of interaction between the polymer and surface, they were used to examine dynamical processes such as the chain dynamics in melts [21,22,23] or the adsorption dynamics [24,25,26] near flat surfaces. The roughness was introduced in generic models by attaching fixed obstacles to a flat surface [27,28,29] or to the walls of a slit [30], which in all cases resulted in reduced lateral mobility of adsorbed polymers with respect to the neat surface. In addition, coarse-grained bead-spring simulations reported higher segmental relaxation times near the rough surfaces with regularly spaced interaction sites [31]. The heterogeneous character of the surfaces was also implemented through a distribution of weakly and strongly interacting sites on a flat wall [32,33].

Atomistic molecular dynamics simulations have been used to describe properties of specific types of hybrid polymer materials, such as graphene-based nanocomposites or silica-filled rubber compounds. This technique captures the chemical details and, thus, describes chemistry-specific polymer–solid interactions. Polymer/graphene or polymer/graphite hybrid materials can serve as a good example of hybrid materials containing flat surfaces [34,35,36,37,38,39,40,41,42,43,44,45,46]. In these studies, the properties are analyzed as a function of the distance from the flat surface, quantifying in such a way the extension of the interphase region, i.e., extension of the region of the altered properties with respect to expected bulk-like behavior of the given polymer. Solar et al. in a series of studies investigated the polybutadiene (PB) films confined by graphite [38,39,40] in which they attributed the dynamical changes near the surface to the interplay of the local density changes near the wall (i.e., enhanced packing near the surface) with the rotational barriers. Pandey et al. [34] performed a detailed spatio-temporal analysis of polyisoprene (PI) on graphite and linked the particular conformation of the adsorbed chain (i.e., train, loop or tail) with its segmental dynamics and its position relative to the surface. The authors reported an intramolecular dynamical heterogeneity for the adsorbed chains; in other words, polyisoprene segments lying on a perfectly planar surface such as graphite differed in dynamics depending on their positions within the train.

Concerning the silica-filled rubber compounds, uniformly dispersed silica nanofillers in cross-linked polybutadiene matrix were simulated in atomistic detail by applying reverse mapping of the well-equilibrated coarse-grained systems [47,48]. Kempfer et al. studied a *cis*-1,4-polybutadiene (PB) film confined between silica planes [49] and nanocomposites composed of the same polymer and filled with bare and grafted silica nanoparticles [50]. The authors based their model of silica on its crystalline β-cristobalite form [49,50], resulting in a regular structure of slab and, thus, also of its boundary atoms [49]. When comparing the behavior of PB chains adsorbed on a highly ordered silica slab and on its annealed and less ordered version, a change in the shape as well as in the positions of the minima in the density profile was observed in the case of the annealed silica surface, pointing out a different packing of the chains near rough surface in comparison to the regular β-cristobalite form. Concerning the amorphous form of silica, a periodic amorphous silica surface in combination with PB polymer chains was chosen to collect information at the atomistic level for the first step of a multiscale methodology [51]. The authors then reported structural and dynamical properties, such as chain dimensions and the relaxation of the end-to-end vector for the chemistry-specific coarse-grained model [51]. Amorphous silica substrates were also used to confine the polyisoprene chains in thin films [52,53]. In their study, Guseva et al. investigated the effect of the film thickness and, thus, of the imposed confinement, reporting mainly the overall properties of the material averaged over the whole film [52]. Recently, conformational and dynamical properties of *cis-* and *trans*-polybutadiene/silica nanocomposites were analyzed by classifying the chains according to their adsorption state and their distance from the nanoparticle’s center of mass [54]. Due to the confinement effects induced by a high volume fraction of the filler as well as due to the curvature of the nanoparticles, a small fraction of PB chains also acted as bridges between the nanoparticles or was wrapped around the filler in addition to trains, loops and tails [54].

Motivated by a recent experimental interest in rough surfaces [11,12,13,14], we aim to provide a detailed analysis of the structure–dynamics relation in hybrid polymer-based materials with irregular interfaces. More specifically, we employ an atomistic molecular dynamics simulation technique to study a realistic model of *cis*-1,4-polybutadiene chains in melt adsorbed on an amorphous silica slab. The planar slab geometry allows us to eliminate the effect of curvature and to collect enough information in the interphase about the interaction of polymer chains with the heterogeneous silica substrate. Films, for which its thickness is sufficient for the chains to recover the bulk-like behavior far from the substrate, are chosen in order to focus on the equilibrium interfacial phenomena without strong confinement effects. We implement an algorithm, which accounts for the irregular surface roughness, in order to analyze the structural and dynamical properties of the film. Consequently, we link local conformational properties with the dynamical response of the material and discuss the similarities and differences with the previously reported interfacial phenomena on flat surfaces. Our results contribute to the complex picture of polymer behavior on solid interfaces, going beyond the (average) density-dynamics relation and pinpointing the fact that the local mobility of the segments is not only dictated by their position with respect to the surface but also that the segments at the same distance from the substrate (and thus in the region of the same density) differ in dynamics due to their local conformation and environment. Insight from the atomistic simulations represents an important asset for the development of multi-scale models [55], as well as for understanding unexpected experimental findings [9,10].

## 2. Model and Simulation Details

Each model system consists of a periodic silica slab and a polymer matrix, namely *cis*-1,4-polybutadiene (cPB), embedded between the slab and its periodic images at 413 K. The polymer chains are either composed of 30 monomeric units (cPB30) or 100 monomeric units (cPB100). The entanglement molecular length of cPB is around 50 monomers, and thus the chosen model systems correspond to a Rouse-like one (cPB30) and a mildly entangled one (cPB100) [56]. The polymer chains are modelled by a united atom model, meaning that the hydrogens are not simulated explicitly but are included together with the carbon atoms in one united atom, e.g., CH. The details of the force field used for cPB can be found elsewhere [54]. Both 30 and 100-mers in bulk were also simulated as reference systems. The model of the amorphous silica is based on the force field of Lopes et al. [57], Ndoro et al. [58] and Pandey and Doxastakis [59]. The slab consists of silicon, oxygen and hydrogen atoms. Only the atoms of the silica slab are partially charged, and the electrostatic interactions are accounted by the reaction field approach. The bonds of hydrogens with oxygens were constrained with the LINCS algorithm [60], and the cut-off distance for the non-bonded interaction potential was set to 2 nm given the absence of long-range dispersion corrections. The details about systems under study are listed in Table 1. Note that the systems are weakly confined, i.e., the film thickness is much bigger than $2R_{g}$.

The systems were prepared and equilibrated via a multiple-step protocol.

**Figure 1:**
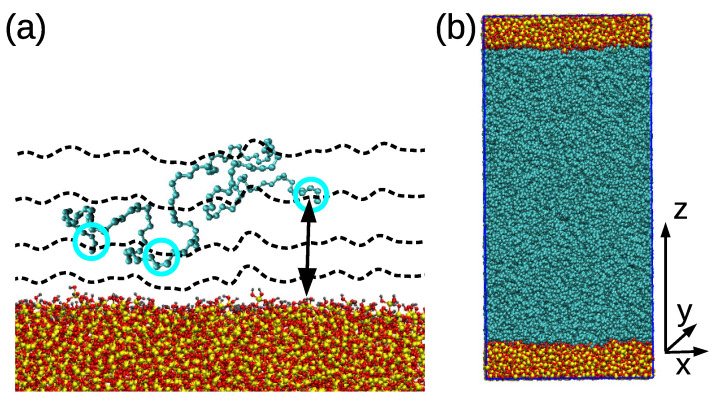
(**a**) Randomly selected chain adsorbed on the silica surface. The cyan circles select a group of atoms used in the monomer-based analysis, and the black arrow denotes the distance criterion used in the algorithm. The dashed lines are drawn to illustrate the space distribution around the substrate after applying the minimum-distance criterion and identifying layers with a width of 0.6 nm. (**b**) Representative snapshot of a well-equilibrated cPB30/Sil system. Cyan color was chosen for PB chains; the slab atoms are yellow (Si), red (O) and grey (H).

1. First, the slab was prepared as follows: An amorphous silica layer containing silicon and oxygen atoms was obtained from a bulk system prepared by following the protocol in [61]. The atoms inside the silica layer were connected according to the method reported in [62]; some of the unsaturated oxygen atoms on the surface were terminated by bonding hydrogen atoms to them.
2. Then, the polymer chains were added in the simulation box via a sequence of 30–60 runs, each of 1 ns with the time step of 0.5 fs. During those runs, the added chains were adsorbed to the surface and integrated into the polymer matrix; thus, the regions of heterogeneous density were eliminated. An example of a randomly selected PB chain adsorbed on the surface is shown in Figure 1a. The temperature was maintained by the stochastic velocity rescaling algorithm and the system was coupled to the Berendsen barostat.
3. Once the film was prepared, the time step was increased to 1 fs and the film was equilibrated at the given temperature for about 50–75 ns. The same barostat and thermostat as in the step two were used.
4. The final step was the pre-production run and this was conducted with the same settings as the production run (see below) for about 25 ns.

During the production run, the Nose–Hoover thermostat and Parrinello–Rahman barostat were used to maintain the temperature at 413 K and the pressure at 1 bar. Silica slab and polymer matrix were coupled to separate thermostats to avoid temperature gradients. The time step was 1 fs. The periodic boundary conditions are applied in all three directions. The length of the production run was 350 ns for the cPB30 film and 450 ns for the cPB100. A snapshot of a well-equilibrated cPB30/Sil system is shown in Figure 1b.

In order to calculate the dynamical properties and properly sample the time scales of the local segmental dynamics, we set up five short runs of 5 ns with the frequency of saving 1 ps and one run of 25 ns with the frequency of saving 5 ps.

## 3. Results

### 3.1. Structure of the Interphase

**Figure 2:**
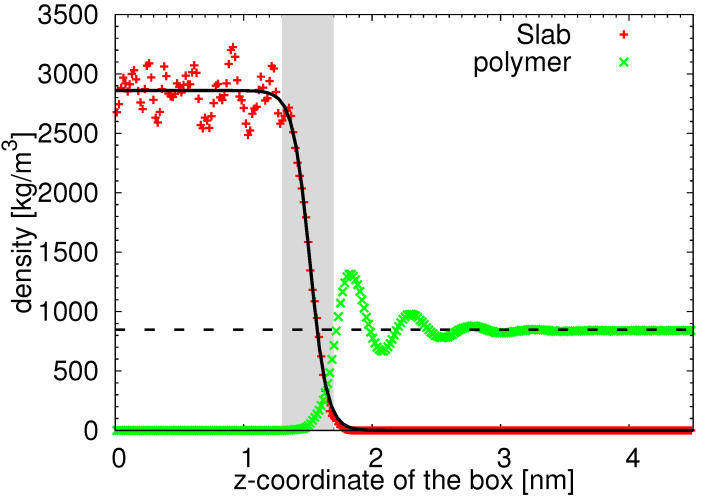
Demonstration of the estimation of the surface roughness. The points represent the atom-based density profiles of two system components of cPB30/Sil system measured along the *z*-axis of the simulation box (see Figure 1). The black solid line is the hyperbolic tangent function used for the profile approximation. The shaded area illustrates the region in the slab vicinity used for the roughness estimation (see the text). The bulk polymer density is shown by the horizontal dashed line.

Before analysing the polymer properties, we turn our attention to the silica substrate. As readily seen in Figure 1a, the surface of amorphous silica is not planar and the silanol groups orient towards the polymer film, resulting in an effective nanoroughness of the solid surface. Notice that these local regions where the surface is uneven are not distributed regularly, as is the case of the crystalline form of silica or coarse-grained models of rough surfaces [27,28,49]. Such a nanoscale roughness is also likely to be found in systems containing functionalized graphene nanofillers, such as graphene oxide [42,43,44], where the nanowrinkling of the graphene contributes significantly to the heterogeneous character of the solid filler in addition to the hydroxyl and epoxide groups attached to the surface. Contrary to 2D materials where the roughness can be estimated as the deviation of “valleys” and “hills” from the optimal plane [46], finding the position of the optimal plane in the irregular structure of the amorphous silica slab studied here is not trivial. In order to avoid this issue, we estimate the roughness of our silica surface from the fit of its atom-based density profile. As readily observed in Figure 2, the atom-based density profile of the silica slab along the *z*-axis of the film is not a step function as it would be in the case of a perfectly planar surface, but it exhibits a smooth decay. We approximate this decay by a hyperbolic tangent function:

(1) $$d\left(z\right)=\frac{\rho_{s}}{2}\left[1-\tanh\left(\frac{z-z_{p}}{2w}\right)\right]$$

where $\rho_{s}$ denotes the average slab density, $z_{p}$ denotes the z-coordinate of the point of inflection of the function and *w* is a measure of the width of the first derivative of the function. The fitting function describes the data very well (compare the solid black line with red symbols in Figure 2). Setting the $z_{p}$ as our reference point, we assume that the boundary atoms of the surface lie in the region of the main decay of the function, i.e., in the region between the two asymptotes. Using the analogy with two-phase systems, the thickness of this region can be estimated as $\delta =4w\tanh^{-1}\left(0.9\right)$ [63,64]. The so-defined boundary region is illustrated in Figure 2 by a shaded area. It is also apparent that the polymer atoms can penetrate this region, as their density is non-zero. Paralleling the experimental studies, where the root mean square roughness $R_{q}$ is measured by atomic force microscopy, we estimate the roughness of our substrate as $R_{q}\approx \delta /2\approx 0.2$ nm. Following another approach used in the X-ray scattering [65], we can assume that the fluctuations around a surface mean height are Gaussian and that the density profile can be either approximated by a hyperbolic tangent function (Equation (2.36) in [65], identical with Equation (1) after the substitution $w=\sigma_{z}\sqrt{3}/\pi$) or by a normalized error function (Equation (2.31) in [65] and Equation (2) below).

(2) $$d\left(z\right)=\frac{\rho_{s}}{2}\left[1-\mathrm{erf}\left(\frac{z-z_{p}}{\sqrt{2}\sigma_{z}}\right)\right].$$

This alternative approach results in $R_{q}\approx \sigma_{z}\approx 0.12$ nm. Both estimations of the roughness in our systems are more than 10 times smaller than the experimentally estimated values of, e.g., plasma-treated silicon wafer substrates [13]. We would like to stress that this approximate estimation of the roughness is indicative of the “ideal” amorphous silica, and we present it in order to demonstrate the absence of deep pores and high obstacles on our surface, which would result in either highly-confined dynamics or a formation of cavities with an excess free volume.

In order to properly account for the non-homogeneous character of the silica interface, we calculate the properties of polymer chains by implementing an algorithm, which defines the distance from the silica slab as the minimum distance of the PB monomer from the atoms of the amorphous silica (see Figure 1a). According to this definition, the monomers, which are at the same distance from the slab, do not form a perfect plane, as it would be in the case of flat surfaces such as graphite. On the contrary, the monomers are aligned in equidistant layers from the surface. The algorithm is applied to both bottom and upper surfaces, finding always the minimum distance to either of them. The measured properties are then averaged for both surfaces. Note that analogous algorithm based on the equidistant layers was also implemented in nanocomposites containing graphene-based nanofillers [43,44,66] and in polymer/silica nanocomposites [59].

**Figure 3:**
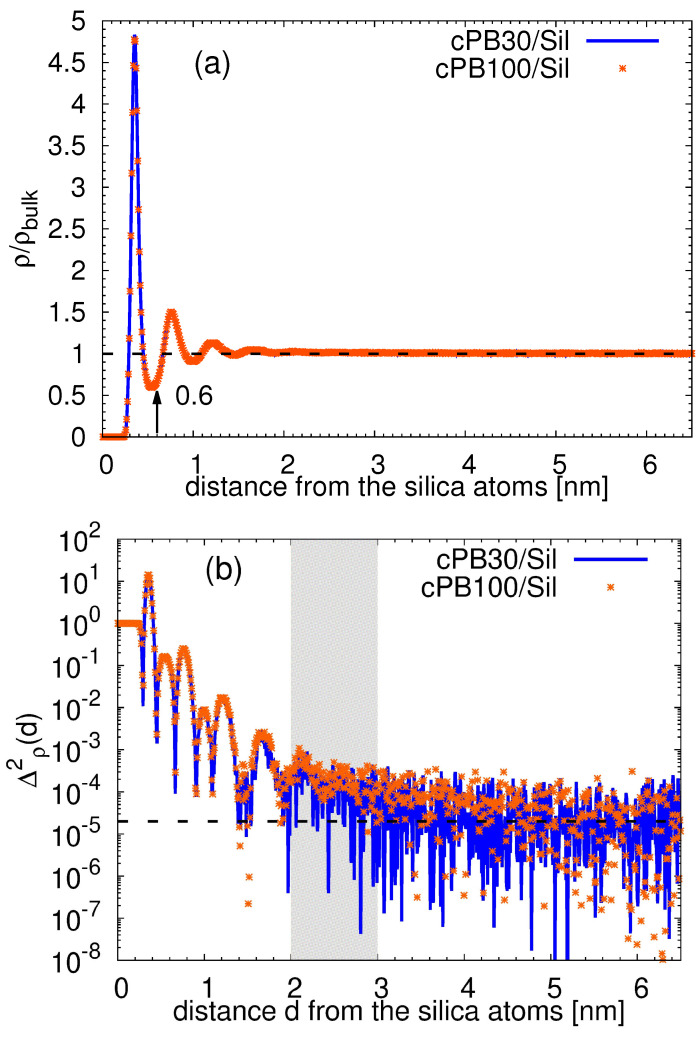
(**a**) Rescaled monomer density profile and (**b**) variance of the rescaled monomer density as a function of the distance from the surface. The arrow in (**a**) points to the first minimum of the function. The shaded area in (**b**) illustrates the range of distances where the variance reaches the plateau.

Having defined the characteristic monomer/substrate distance, we look at a specific alignment of the monomers in each layer. As observed in the monomer density profile in Figure 3a, the packing of the monomers near the silica slab is tighter than in the corresponding bulk, giving rise to a well-distinguished maximum in the density profile. This feature is typical for surfaces with favorable effective interactions with polymer. The position of the successive minimum is usually taken as a first estimation of the width of the first adsorbed layer. The monomer density profiles of both studied polymers cPB30 and cPB100 are identical, and their first minimum is placed at the distances around 0.6 nm. However, closer inspection of Figure 3a reveals that there are perturbances in density at distances larger than 0.6 nm. In order to quantify the extent of this effect, we calculated the variance $\Delta^{2}\rho \left(d\right)$ of the density. More specifically, $\Delta^{2}\rho \left(d\right)={\left(\langle \rho (d)\rangle -\rho_{\mathrm{bulk}}\right)}^{2}$, where $\rho_{\mathrm{bulk}}$ is the average density of the bulk, and *d* denotes the distance from the silica slab. The same quantity has been used in the simulation study which employed the bead-spring model to examine the chain stiffness effect on the properties of chains adsorbed on a flat surface; there, the authors assigned the saturation point of $\Delta^{2}\rho \left(d\right)$ vs. *d* to the transition point from the perturbed to the unperturbed (bulk-like) behavior [17]. The variance as a function of the distance *d* is plotted in Figure 3b. Similar to the observation in Figure 3a, also $\Delta^{2}\rho \left(d\right)$ of both polymers seems to be identical. We estimated the transition point to be at the distance d≈ 2–3 nm (shaded area in Figure 3b) from the surface for both studied polymers. Note that this result also confirms that the thickness of the studied films is sufficient to observe a bulk-like behavior of the chains in the middle of the film.

**Figure 4:**
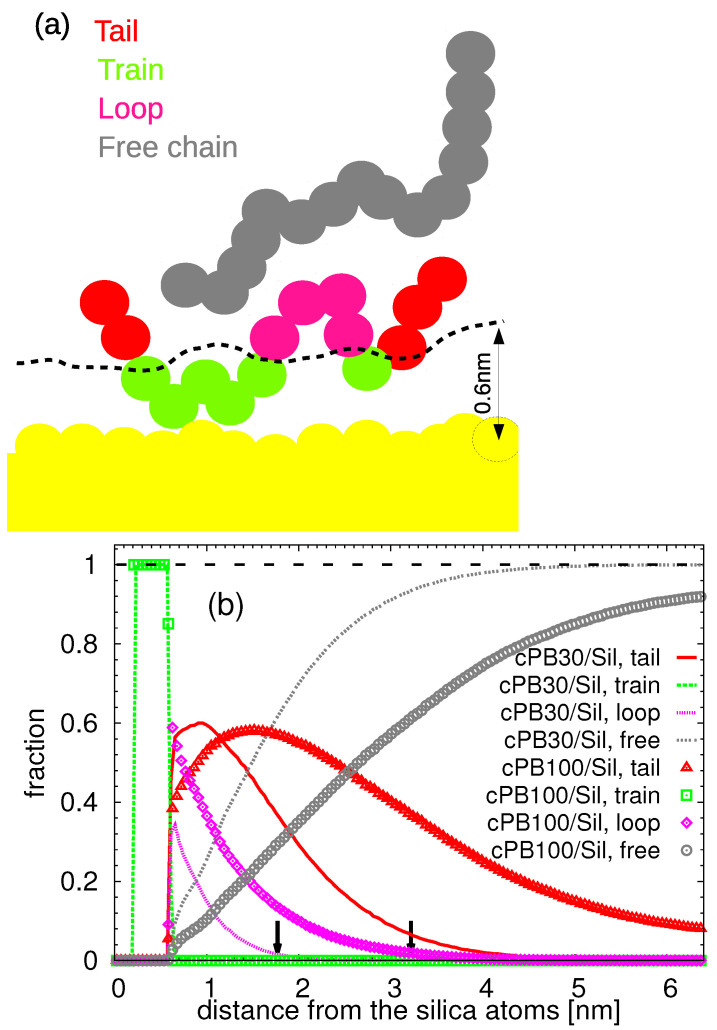
(**a**) Schematic illustration of the conformations of the adsorbed chains. (**b**) Fraction of the given conformation as a function of the distance from the slab. Small black arrows denote the the distance of 0.5$R_{e}$ from the substrate, with $R_{e}$ being the average end-to-end distance of the cPB30 chains (left arrow) and of the cPB100 chains (right arrow) in the bulk.

In Figure 4a, a schematic illustration of chain conformations at the cPB/silica interface is shown: the train conformation, which comprises only adsorbed monomers; the loop conformation, which is the part of the chain between two adsorbed monomers; and the tail one, which is characteristic for the chain ends of adsorbed polymers. Here, we consider a monomer to be adsorbed when its distance to the silica slab is smaller than 0.6 nm, which corresponds to the first minimum in the density profile (see Figure 3a). Chains for which its monomers are not adsorbed are labelled as free. Such a geometric criterion has been widely used in the literature as well. Note that the adsorbed region can be also defined as the region where the polymer dynamics deviate from those in bulk (see the discussion in the following section). We avoided labeling monomers according to their dynamics near the surface, e.g., according to their characteristic relaxation time, since choosing a specific characteristic time from the continuous distribution of residence times would be rather arbitrary. Note also that in what follows, we present results that do not depend on this geometric criterion in order to examine various interpretations of adsorbed region.

The fraction of each conformation in the equidistant layers parallel to the surface is plotted in Figure 4b. By definition, at the nearest neighbor distances up to 0.6 nm relative to the silica slab, only train conformations are present. As the distance from the slab increases, the tail conformation prevails for cPB30 chains up to d≈1.5 nm, and the film is then mostly composed of free and non-adsorbed chains. As expected, the loops are much more populated in cPB100 systems, in contrast to the cPB30, given its higher molecular weight and, thus, higher number of monomeric units which can “interconnect” two train conformations. Moreover, at very short distances, much smaller than the radius of gyration of cPB100 chains (see Table 1), the loop conformations dominate over tails, as theoretically expected for much longer chains [6,7]. Consequently, the extension of the so-called bound loop layer changes with molecular weight. The bound loop layer represents the region consisting of the loops of the adsorbed chains, and it has been suggested that its width is related to the width of the region with altered dynamics [9,10,67]. As readily observed in Figure 4b, the fraction of the loops decays to 0 around 2 nm in the case of cPB30 and around 3 nm in the case of cPB100 (notice the decay of the magenta ultrafine dashed line and magenta squares in Figure 4b). It is important to note that in contrast to the experimental observation in the systems with up to 20 vol % loading of silica nanoparticles [9], we do not observe any difference in the monomer density for the two studied polymers (see Figure 3a) in the bound loop layer; the loops in our systems are neither confined nor in a metastable state. When rescaled with the end-to-end distance of the bulk chains, $R_{e}$, both bound loop layers seem to be extended up to a distance equal to 0.5$R_{e}$ from the substrate.

**Figure 5:**
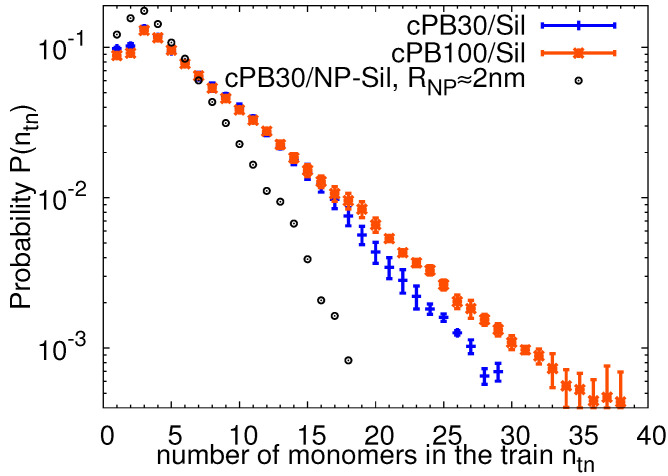
The probability distribution function of finding a train consisting of $n_{\mathrm{tn}}$ monomers. The error bars were obtained by a standard block averaging method. For comparison, the data obtained for nanocomposites filled with silica nanoparticles (30 wt %) with a radius of approximately 2 nm are reported. The details about the nanocomposites can be found in Reference [54].

Considering the conformational changes of the polymer chains in the adsorbed layer, i.e., the conformational behavior of trains, we measured the probability of finding a train configuration consisting of a given number $n_{\mathrm{tn}}$ of monomers. The probability distribution functions for both studied polymer lengths are plotted in Figure 5. The distributions match within the accuracy in the region of low $n_{\mathrm{tn}}$, both exhibiting a maximum at $n_{\mathrm{tn}}=3$ monomers. The position of the maxima is in agreement with the maxima found for indentical cPB chains adsorbed on curved surfaces, namely on the amorphous silica nanocomposites (compare the crosses with circles in Figure 5 and see [54] for details about the nanocomposite system). Concerning the shape of the distribution, shorter trains are preferentially formed in the nanocomposite system in comparison to the planar slab surface where the effect of the curvature is eliminated. Interestingly, when atoms instead of monomers are used for the train characterization, a slightly shifted maxima was reported for planar and curved surfaces in [59], where the authors compared the behavior of polyethylene chains interacting with an amorphous silica slab and with spherical silica nanoparticles. It is important to note that the shift was observed for the nanocomposite systems where the radius of the nanoparticle was comparable to the Kuhn length of the polymer. The data presented in Figure 5 correspond to a system in which the radius of nanofillers is around two times bigger than the Kuhn length of the polymer matrix (see [54] for the estimation of the Kuhn length for the used PB model). The probability of finding trains formed by the whole chain, i.e., for which its length equals to the length of the polymer chain, is higher in the systems with shorter cPB30 chains than cPB100 due to the lower entropic penalty associated with the full-chain adsorption in the former case, as also reported in the literature for flat surfaces [35] and silica nanocomposites [54].

### 3.2. Dynamics at the Interface

**Figure 6:**
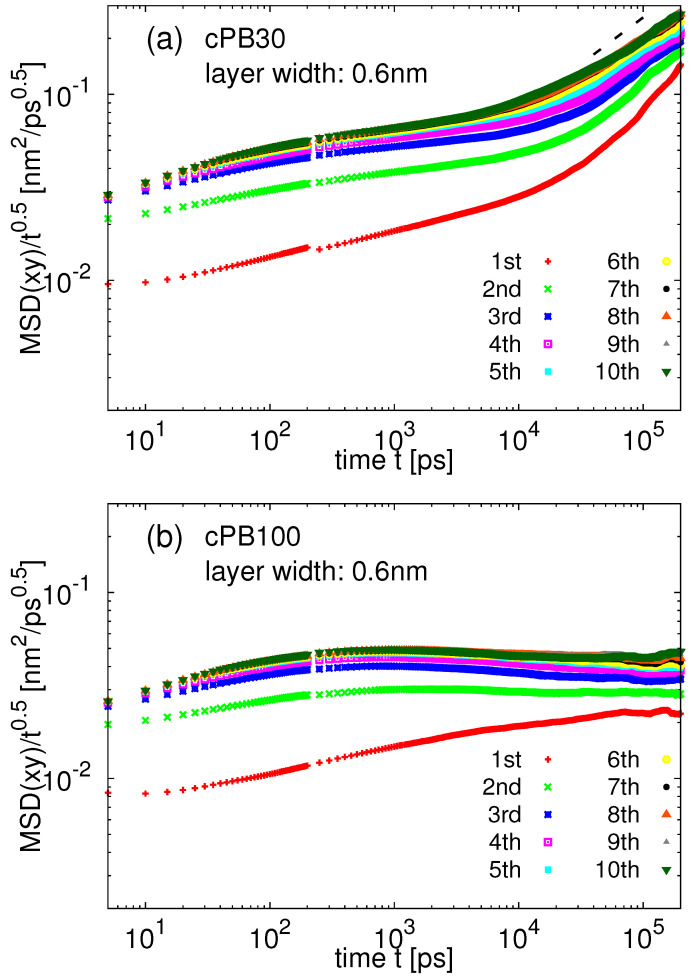
Monomer MSD in the direction parallel to the surface normalized by the square root of time, $t^{0.5}$, for (**a**) cPB30/Sil and (**b**) cPB100/Sil system. The width of each layer is 0.6 nm. The black dashed line in (**a**) is guide for the eye and represents the scaling in the terminal diffusive regime, MSD $∼t^{1.0}$.

In order to explore the effect of the rough silica surface on the translational motion of the cPB monomers in its vicinity, we calculated the monomeric mean squared displacement (MSD) in the direction parallel to the surface (xy in Figure 1). Namely, we divided the space in the vicinity of the slab into layers of width 0.6 nm and measured the motion of the monomers in these layers. The layer was assigned in the beginning of the measurement, and thus the monomers can freely diffuse between the layers during the measurement. Note that if we restricted our calculation only for the monomers, which stay in the same layer during the whole duration of the measurement, the measured displacement would depend on the frequency of saving the trajectory frames. The time evolution of the monomeric MSD normalized by the square root of time, $t^{0.5}$, in different layers is plotted in Figure 6a for the cPB30/Sil system and in Figure 6b for cPB100/Sil system. This representation facilitates the detection of the Rouse-like dynamics, which predicts that the segmental dynamics follows the MSD $∼t^{0.5}$ scaling in the short time regime before approaching the terminal diffusive dynamics. Both polymers show deviations from the bulk-like behavior in the layers near the slab at early and intermediate times. More specifically, in both Figure 6a,b, the first three layers are slower than the layers in the middle of the film (labels fifth and higher). In other words, the monomeric translational motion in the direction parallel to the surface is affected up to d≈ 2 nm in the case of both studied molecular weights of cPB. This effect is vanishing as the chains reach the terminal diffusive regime, which can be detected as an upturn in Figure 6a at the end of the simulation time window. The mildly entangled cPB100 chains do not enter the terminal diffusion regime within the simulation time window. These chains exhibit Rouse-like dynamics with a characteristic MSD $∼t^{0.5}$ behavior up to $t\approx 10^{4}$ ps, where a slightly negative slope can be observed for segments in the middle of the film (labels fifth and higher). A negative slope in this representation indicates the presence of entanglements [68], as also shown in our recent study of bulk PB [69].

Very recently, mobility in the direction parallel to the surface was studied by means of atomistic molecular dynamics simulation in the systems of polystyrene and poly(acrylic acid) on the pristine graphene and graphene oxide [42]. The authors observed that the mobility deviated from the bulk-like behavior more significantly in the case of the rough, graphene oxide surface than in the case of the pristine graphene. They partially attributed this effect to the formation of hydrogen bonds between the grahene oxide and the polymer matrix [42]. This is not the case in our systems, and thus the decreased lateral mobility of the monomer is most probably associated with the roughness of the amorphous silica slab and with the relatively strong adsorption of the polymer on it (see the intensity of the main peak in Figure 3a). The same phenomenon of slower lateral diffusion was also observed in bead-spring polymer models on strongly adsorbing flat surfaces [23]. Note that negligible alteration of the lateral mobility was found for chains on graphite and pristine graphene, which can be considered as perfectly planar [36,45].

**Figure 7:**
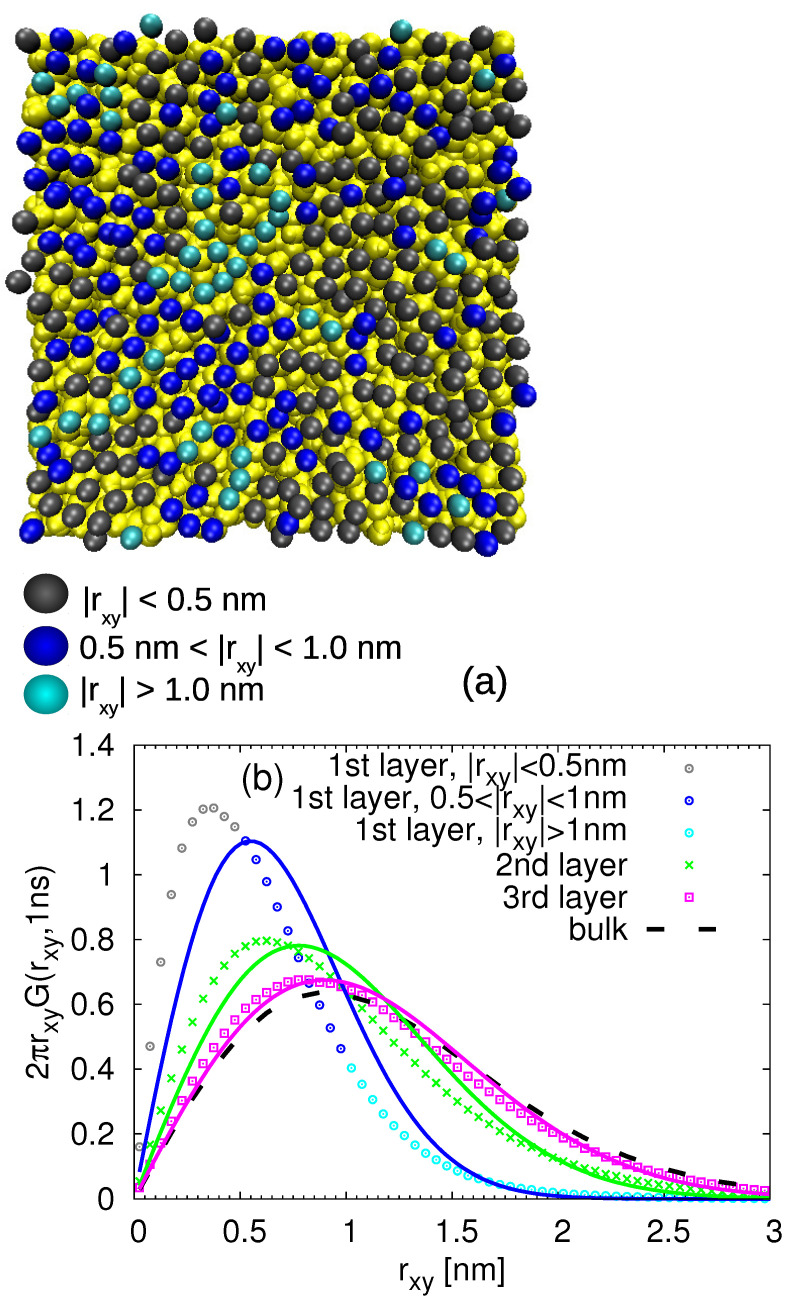
(**a**) Snapshot of the adsorbed monomers of cPB30 highlighted with different colors according to their mobility during 1 ns. The atoms of the substrate are yellow. (**b**) Van Hove function including lateral displacement of the monomers in the first 4 layers (the first layer corresponds to the adsorbed one) for t=1 ns. The solid lines are the Gaussian distributions with the lateral MSD taken from the simulation.

**Figure 8:**
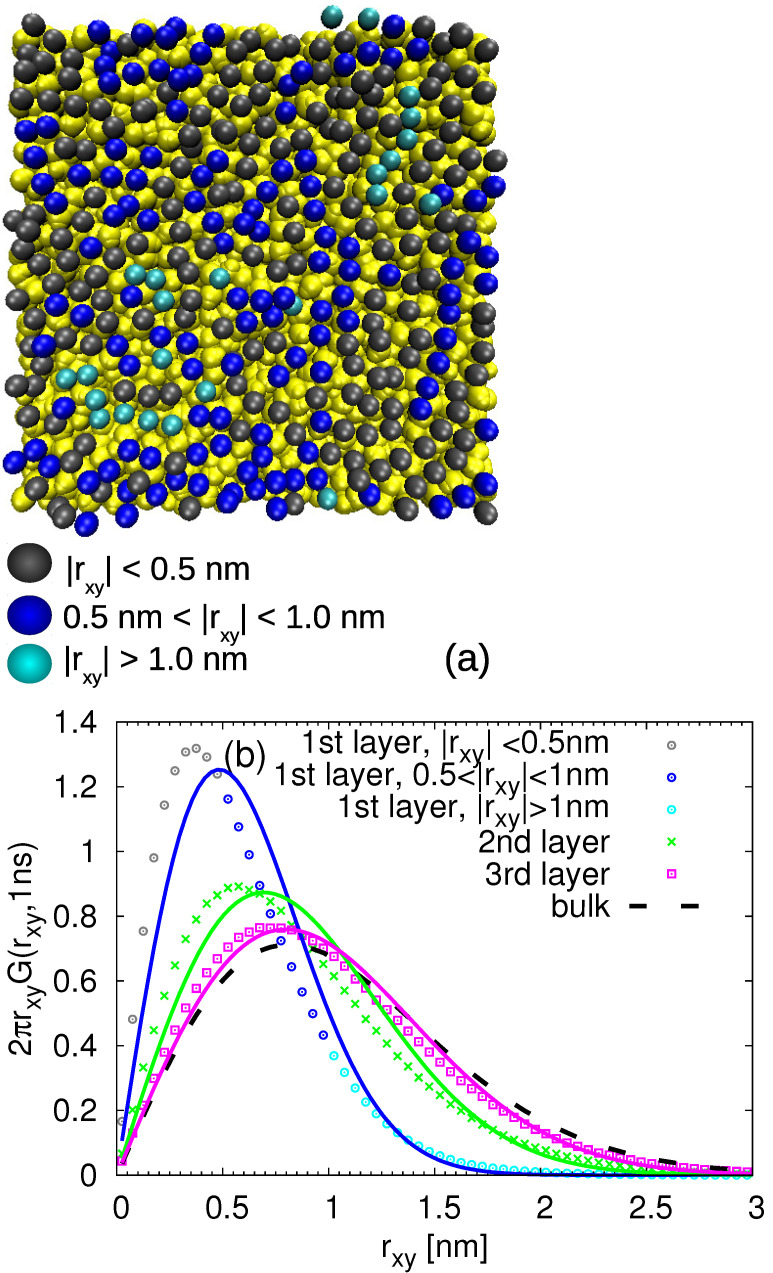
(**a**) Snapshot of the adsorbed monomers of cPB100 highlighted with different colors according to their mobility during 1 ns. The atoms of the substrate are yellow. (**b**) Van Hove function including lateral displacement of the monomers in the first 4 layers (the first layer corresponds to the adsorbed one) for t=1 ns. The solid lines are the Gaussian distributions with the lateral MSD taken from the simulation.

In order to distinguish the slow and fast populations of the monomers in each layer, we first started with the visual inspection. More specifically, we labelled all the monomers within the distance 0.6 nm from the silica slab and then we monitored their displacement parallel to the surface in the time window of 1 ns. This time is chosen to explore the intermediate times at which the monomer moves multiple times its size (i.e., approximately five times the monomer end-to-end distance) but before it reaches the diffusive regime (see Figure 6). Then, we highlighted the monomers according to their mobility with different colors. The representative snapshots of this procedure are shown in Figure 7a and Figure 8a. It is obvious that, even within the given layer, there is diversity in the mobility of the selected monomers. However, the pattern and arrangement of the slow and fast populations of the monomers seems to be random at 413 K (much above the glass transition temperature, $T_{g}$, of cPB).

In order to quantify the deviations from the Gaussian behavior, we calculated the Van Hove function G(r,t) for monomers in each layer as follows:

(3) $$G\left(r_{\mathrm{xy}},t\right)=\frac{1}{M_{i}}\langle \sum_{i=1}^{M_{i}}\delta \left(r_{\mathrm{xy}}+{r_{\mathrm{xy}}}^{i}\left(0\right)-{r_{\mathrm{xy}}}^{i}\left(t\right)\right)\rangle$$

with $M_{i}$ being the total number of monomers in the given layer and $r_{xy}$ denoting the displacement parallel to the surface. The obtained $G(r_{\mathrm{xy}},t)$ functions were further confronted with the Gaussian-like behavior:

(4) $$G\left(r_{\mathrm{xy}},t\right)={\left(4\pi D\left(t\right)t\right)}^{-1}\exp\left(-\frac{{\left(r_{\mathrm{xy}}\right)}^{2}}{4D(t)t}\right)$$

where D(t) denotes a time-dependent diffusion coefficient parallel to the surface calculated at the given time t=1 ns as $D\left(t\right)={\mathrm{MSD}}_{\mathrm{xy}}\left(t\right)/4t$, with MSD${}_{xy}\left(t\right)$ being the monomeric mean square displacement in the given layer (see Figure 6) at this time. The so-obtained quantities are plotted in Figure 7b for the cPB30/Sil system and in Figure 8b for the cPB100/Sil system. In agreement with the observations related to Figure 6, the Van Hove functions seem to be shifted to smaller $r_{xy}$ values in the first three layers in comparison to the bulk, clearly reflecting the slower mobility parallel to the surface in these regions. The first two layers (up to d≈1.2 nm) also exhibit clear deviations from the Gaussian behavior (compare symbols with solid lines in Figure 7b and Figure 8b), however, the deviations in the third layer are marginal, and the $G(r_{\mathrm{xy}},t)$ in this particular layer follows the Gaussian behavior pretty well.

**Figure 9:**
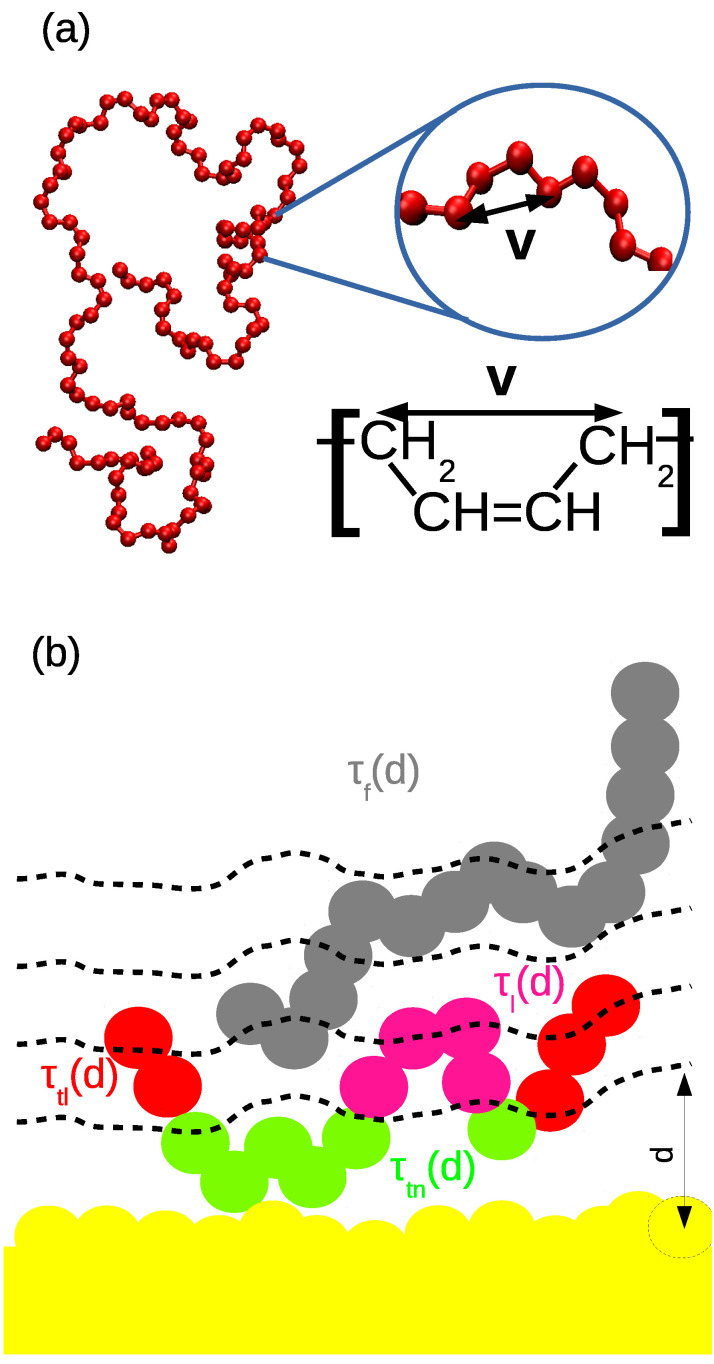
Schematic illustration of the (**a**) 1–4 vector along the backbone and (**b**) dependency of the characteristic times on the layer position and on the classification of the polymer configuration ($\tau_{l}\left(d\right)$ stands for the characteristic time of a monomer at distance *d* belonging to a loop, while $\tau_{\mathrm{tl}}\left(d\right)$, $\tau_{\mathrm{tn}}\left(d\right)$ and $\tau_{f}\left(d\right)$ correspond to tails, trains and free chains, respectively).

**Figure 10:**
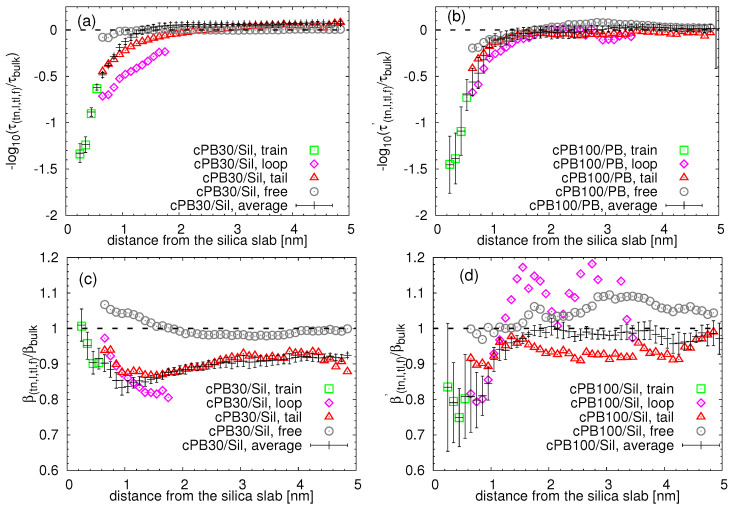
(**a**,**b**) Characteristic correlation times for the trains, loops, tails and free chains as a function of the distance from the slab divided by the same quantity for the bulk. (**c**,**d**) Stretch exponents related to (**a**,**b**).

Concerning the local dynamical heterogeneities in rotational dynamics, we focus on the decorrelation of the vector along the backbone in the vicinity of the amorphous silica. The first Legendre polynomial (correlation function) was calculated for the vectors v along the backbone connecting the first and the last united atom in the monomer (i.e., 1–4 vector, see Figure 9a). In the case of the unentangled cPB30 chains the correlation function exhibits one main decay, which can be approximated by a single stretch exponential function (see Figure S1 in Supplementary Materials). On the other side, an additional, slow relaxation mode contributes to the correlation function of slightly entangled chains in cPB100/Sil system. In order to capture that, we fitted the correlation function in the cPB100/Sil system by a sum of two stretch exponential functions (see Supplementary Materials for more information; examples of the fitting procedure can be found in Figure S1). In this manner, we obtain characteristic time for each type of configuration $\tau_{i}$, with “i” being a label denoting “tn” (train), “l” (loop), “tl” (tail) and “f” (free) segments, as a function of distance from the rough surface (see Figure 9b) as well as the average segmental time. The layer assignment and the classification of the configuration (i.e., train, loop, tail and free) were performed in the beginning of each measurement; thus, monomers can rearrange their positions within the examined time window. The results of this analysis are summarized in Figure 10. Note that to have a fair comparison between the two systems, we report the relaxation time corresponding to the main primary decay of the correlation function in the case of 100-mer PB, i.e., the relaxation time of the slow modes is not considered in Figure 10 (the data are reported in Figure S2 of Supplementary Materials). Moreover, to highlight the different trends in dynamical heterogeneities (represented by the stretch exponent $\beta_{i}$), we present the two parameters obtained from the fits of correlation functions separately (i.e., $\tau_{i}$ and $\beta_{i}$), in contrast to some previous works where the average (integrated) version of the relaxation time has been chosen.

There are two common features for all adsorbed configurations and for both studied chain lengths apparent from the plots in Figure 10a,b. The first one is the increasing tendency of the relaxation times with the decreasing distance from the slab; in other words, the segmental relaxation becomes slower as the segments approach the silica slab (reflected by lower values of $\tau_{\mathrm{tn},l,\mathrm{tl},f}/\tau_{\mathrm{bulk}}$ in the negative logarithmic representation in Figure 10a,b). This deviation from the bulk behavior is present at distances lower than approximately 2 nm from the substrate, in agreement with the previously discussed deviations in translational dynamics (notice the deviation of black points and black dashed line in Figure 10a,b). The second one is a broad spectrum of relaxation times for each adsorbed configuration, i.e., for trains, loops and tails. In the bound loop layer, i.e., at the distances bigger than 0.6 nm and smaller than 0.5$R_{e}$(bulk), the loop segments are always the slowest, owing to their double “contact” with the substrate. The tail segments relax slightly faster than the loop segments, but are generally slower than the bulk. The dependence of the monomer relaxation time of the free chains on the distance from the substrate is negligible for both cPB30 and cPB100. Very similar observations were made in the systems of polyisoprene adsorbed on graphite [34]; however, the actual quantitative differences among the relaxation times of train, loop and tail conformations were slightly smaller in the graphite systems for the short unentangled chains.

Interestingly and in contrast to the results reported for graphite and pristine graphene in References [34,36,45], a non-monotonic dependence on the distance from the silica slab was found for the stretch exponents associated to the relaxation times. Generally, the stretch exponent encaptures the broadness of the distribution of the relaxation times for which the mean value is represented by τ. Low values of β are characteristic for systems with heterogeneous dynamics. Despite the considerable error bars particularly in the cPB100/Sil systems (see Supplementary Materials for an extended discussion about the origin of the error), we found the non-monotonic dependence in both systems. Keeping in mind that the most heterogeneous character reflected by the lowest values of the stretch exponent is mostly observed in the closest vicinity of the surface, most specifically in the region around the first adsorption layer (i.e., at distances between 0.6 nm and 1 nm), we attribute these heterogeneities to the adsorption dynamics and the diffusive movement between the layers adjacent to the silica slab. In other words, as our analysis scheme allows for the motion between the layers (i.e., we assigned the layer in the beginning of the measurement), the dynamics of the monomers placed around 0.6–1 nm and measured by our protocol may be a combination of dynamics of “just-desorbed” monomers with those that are about to be adsorbed and, thus, coming from a slightly faster region. Recently, the same analysis protocol was applied in the systems of poly(methyl methacrylate) confined between pristine graphene, and the authors also reported a non-monotonic dependence of the stretch exponent on the distance from the graphene [44]. Moreover, comparing the two chain lengths, the non-monotonic tendency of β seems to be more marked in the cPB30/Sil system. Considering the above-mentioned hypothesis, the more heterogeneous features in cPB30/Sil would be in agreement with the observed more pronounced deviation from the expected Gaussian behavior in the layers adjacent to the substrate in Figure 7 in comparison to Figure 8.

**Figure 11:**
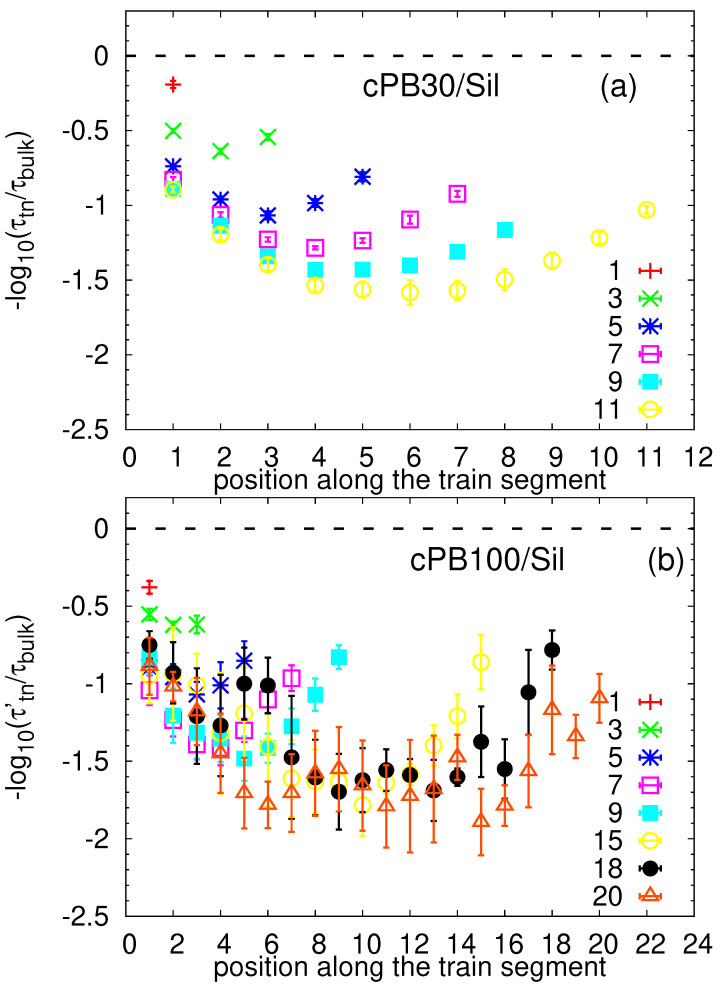
Relaxation times for the segments along the train for trains of different length for (**a**) cPBB30/Sil and (**b**) cPB100/Sil normalized by the corresponding bulk values. The error bars were obtained by averaging over results from five independent runs.

Motivated by the segmental dynamical heterogeneities detected in Figure 10, we turn our attention to the internal dynamical behavior of train segments. First, we located all monomers in the adsorbed layer, i.e., with a minimum distance to the silica atoms smaller than 0.6 nm. Then, we labelled the segments with the numbers in sequential order according to their position in the train, i.e., the first segment was labelled with one, the second with two and so on, and the last segment had a number identical with the number of monomers in the given train. Consequently, we calculated the correlation functions for each 1–4 vector and averaged them over the same positions in trains. Similarly to the analysis presented above, the correlation functions were fitted by stretched exponential functions to obtain the characteristic time for each train segment (see Supplementary Materials for more information and Figures S3 and S4 for examples of the fitting procedure). The relaxation times for different train lengths are plotted in Figure 11. The error bars were obtained as the standard deviation from five independent runs.

Comparing the two studied systems, the relaxation times for the trains of the same length in the cPB30/Sil and cPB100/Sil systems are very consistent, i.e., the data normalized by the corresponding bulk values overlap within the error bars for the majority of the train segments. Interestingly, focusing on the data for particular train configurations, the relaxation times exhibit a non-monotonic dependence on the position of the segment in the train sequence. More specifically, the terminal monomers in the train seem to relax faster than the internal train segments. The distributions appear to be symmetric with respect to the central monomer of the train, and the observed minor deviations from a perfectly symmetric function are most probably due to the statistical fluctuations. One possible explanation for the dynamical difference between the inner and the outer monomers would be a preferential adsorption of the inner train segments in “valleys” and of the terminal segments on “hills” on the rough surface where they have more configurational freedom [70,71]. However, since an internal dynamical heterogeneity in trains has been reported before for polyisoprene chains adsorbed on a graphite surface [34], which in contrast to graphene does not tend to wrinkle [46], we attribute the observed behavior to the entropic effects due to the terminal position in the train and not due to the spatial position on the rough surface. More specifically, as only one of the adjacent monomers of the terminal segments are actually adsorbed on the surface, the second one belongs to the more mobile tail or loop (see Figure 10) and the terminal segment is more prone to desorb, which facilitates its 1–4 vector relaxation. The fact that the fastest dynamics is found for the train with the length of only one monomer, resulting in both neighbors belonging to either tails of loops, is corroborating our hypothesis. Moreover, it seems that this effect is propagated along the polymer via chain connectivity, i.e., the orientational relaxation of the terminal segments is correlated with the adjacent segments, speeding up their dynamics, which results in slightly faster dynamics of the connecting segments. As a result, a symmetric dependence of the relaxation on the position of the segment in the train configuration is observed.

In the long trains, the enthalpic contribution (i.e., increased number of contacts with the surface) and the connectivity effect wins over the entropic one, and the dynamics becomes slower as the length of the train increases (notice the difference in the actual values of $\tau_{\mathrm{tn}}$, e.g., for trains consisting of 9 and 3 segments in Figure 11). By contrast, the segments of the shortest trains (up to three monomers, i.e., up to the Kuhn length [54]) are even slower than the first (terminal) monomers of the longer trains. Due to the low probability of finding long trains (see Figure 5), we cannot obtain statistically relevant results for much longer trains than those presented in Figure 11. However, a closer inspection of the longest trains in Figure 11b reveals that entropic factors are propagated up to the sixth monomer from the train extremes, and the relaxation time of the inner segments reaches a plateau for trains longer than 15 monomers. The trains with 20 monomers relax the inner segments at the same time as the 15-mer and 18-mer trains; therefore, we speculate that for trains longer than (6 + 6) monomers, the entropic and enthalpic contributions are balanced, and their dynamical profiles of $\tau_{\mathrm{tn}}$ are similar, with the only difference being the extended plateau region for inner segments. Note that 12 monomers correspond to approximately 5 Kuhn lengths [54].

**Figure 12:**
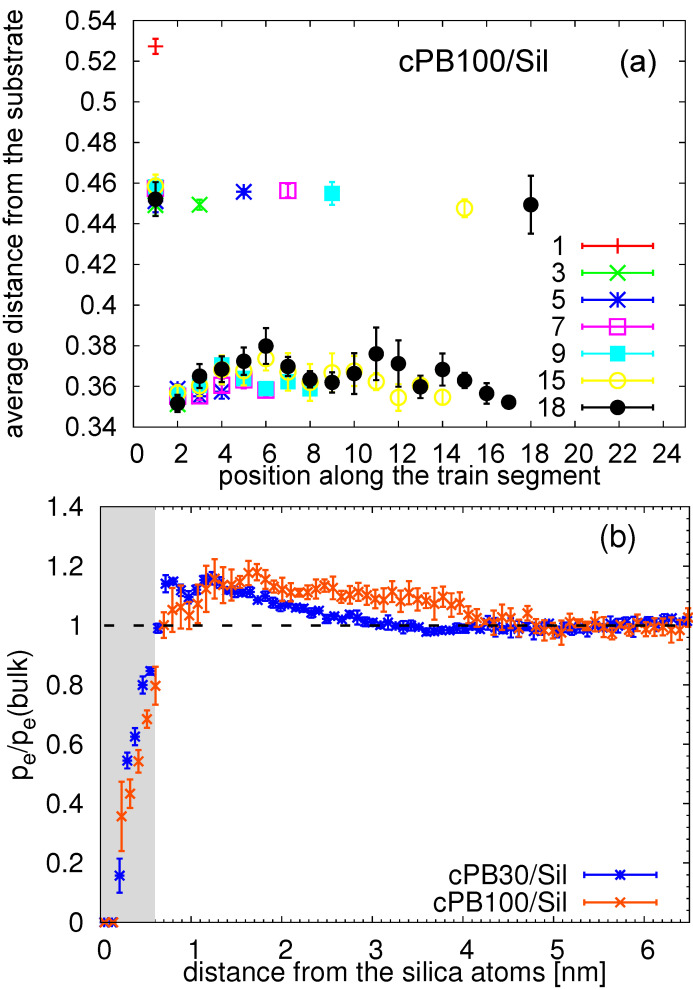
(**a**) Average distance from the substrate (in nm) for the train segments as a function of the position in the train for cPB100/Sil. (**b**) Percentage of the end monomers $p_{e}$ as a function of the distance from the surface normalized by the percentage of the end monomers in the bulk layer $p_{e}$(bulk). The shaded area highlights the region occupied by the train segments. The error bars were obtained by the standard block averaging method.

Linking these observations to those from Figure 10, one may obtain an impression that the difference in dynamics of the train segments may be caused by their different distance from the substrate, or in other words, that the long trains are slower because they are positioned closer to the silica slab. This is not the case, as can be observed also in Figure 12a and Figure S5 of Supplementary Materials. Only the terminal train monomers are located slightly further away from the surface, as expected also for perfectly planar surfaces [35], because they serve as “connecting points” for loops and tails; the remaining inner train segments share the same average distance from the substrate. Therefore, it is clear that the actual polymer/interface distance contributes to the dynamical gradient along the trains observed in Figure 11 only marginally.

Since the chain ends exhibit greater free volume than the inner parts of the chain, we also investigate the relation between the terminal segments in trains and terminal segments in chains. In other words, we examine the possibility that the faster dynamics observed for the terminal segments in train is caused by chain-end effects. To this end, we calculate the percentage of the end monomers with respect to all monomers, $p_{e}$, in the region near the surface. In order to make a fair comparison between the two studied polymer lengths, we normalized the quantity with the percentage of the end monomers in the bulk-like region, $p_{e}$(bulk). As seen in Figure 12, the nearest neighbor regions of the amorphous silica are poor in chain ends for both cPB30/Sil and cPB100/Sil systems, pointing out the fact that the inner parts of the chains are preferably adsorbed on the surface. More specifically, we quantified that only 7.5% and 2.1% of the terminal train segments correspond to the actual chain end (i.e., to the last monomer in the chain) in cPB30/Sil and cPB100/Sil systems, respectively. Therefore, the difference in the dynamics of terminal train segments observed in Figure 11 is not caused by the chain-end effects, as the vast majority of terminal train segments are inner chain monomers. Furthermore, the quantity presented in Figure 12 reaches values slightly higher than 1 in regions near the surface which correspond to the regions with the highest population of tails (see Figure 4b). Note that similar quantity as the one in Figure 12 was reported in Reference [52], revealing a deficit in terminal CH3 groups of polyisoprene in the vicinity of the amorphous silica surface.

**Figure 13:**
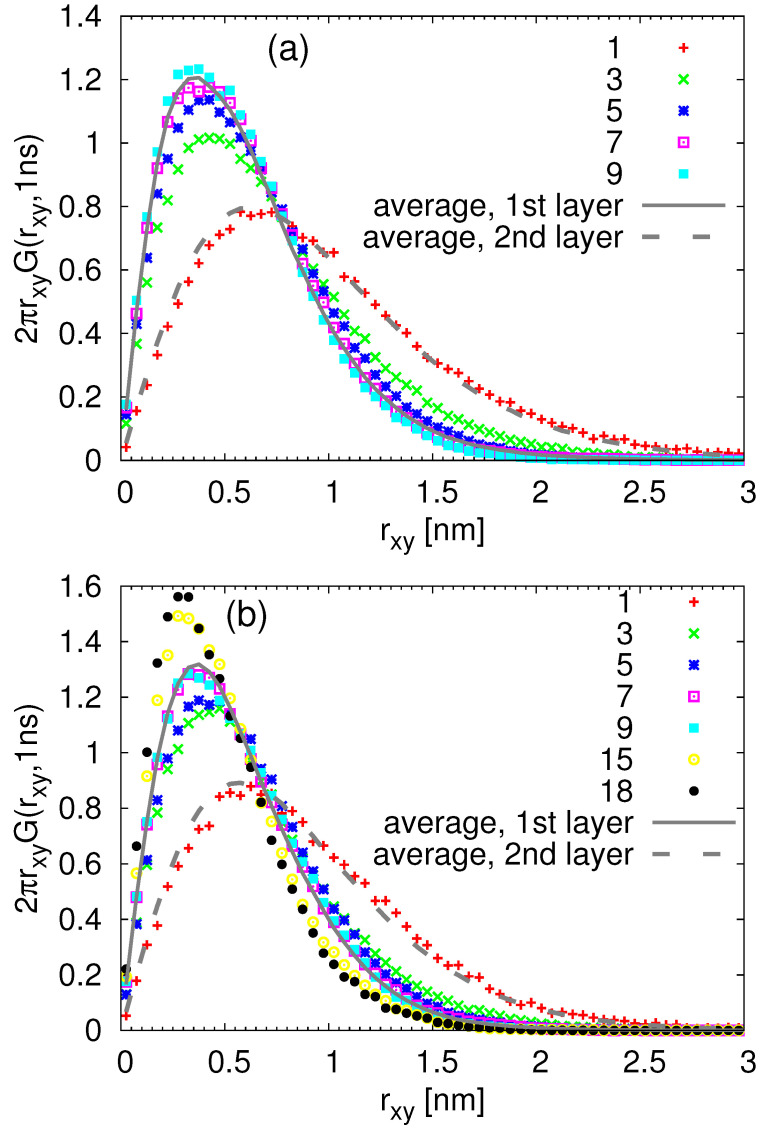
Van Hove functions including lateral displacement of the train monomers for t=1 ns for the (**a**) cPB30/Sil and (**b**) cPB100/Sil system. The legend denotes the length of the trains (i.e., the number of monomers in each train). The lines show the data identical to those presented in Figure 7b and Figure 8b.

In order to relate the above observations of internal heterogeneities in orientational relaxation of train segments with their translational dynamics, we return to the analysis presented in Figure 7 and Figure 8. Namely, we selected the train monomers and calculated the Van Hove functions at time t=1 ns for different train lengths. The results are presented in Figure 13 for both studied chain lengths. With this detailed insight on how the trains of different lengths participate in the dynamics of the (first) adsorbed layer, it is clear that the overall Van Hove function for the adsobed layer (shown in Figure 7 and Figure 8 and in Figure 13 by solid line) does not follow the Gaussian-like trend due to the different lateral mobilities of the contributing monomers. More specifically, in agreement with the observations made for rotational dynamics, the short trains of less than 3 segments exhibit faster lateral diffusion than those with more adsorbed monomers. Moreover, the Van Hove functions for very long trains in the cPB100/Sil system shown in Figure 13b are very narrow, indicating a highly restricted lateral motion. Interestingly, the distributions of the lateral displacement for trains with only one monomer are almost identical with the Van Hove functions averaged over all the monomers placed in the second layer, i.e., in the distance 0.6–1.2 nm from the substrate atoms (compare the red points and dashed grey line in Figure 13a,b). As such short trains are very prone to desorb due to the prevailing entropic effects, it is very likely that they diffuse to the second layer where they become a part of a tail or a loop. These configurational forms show faster dynamics than trains (see Figure 10), which would explain the shift of the Van Hove functions of the trains “traveling” between the first and the second layer towards higher values of lateral displacement.

**Figure 14:**
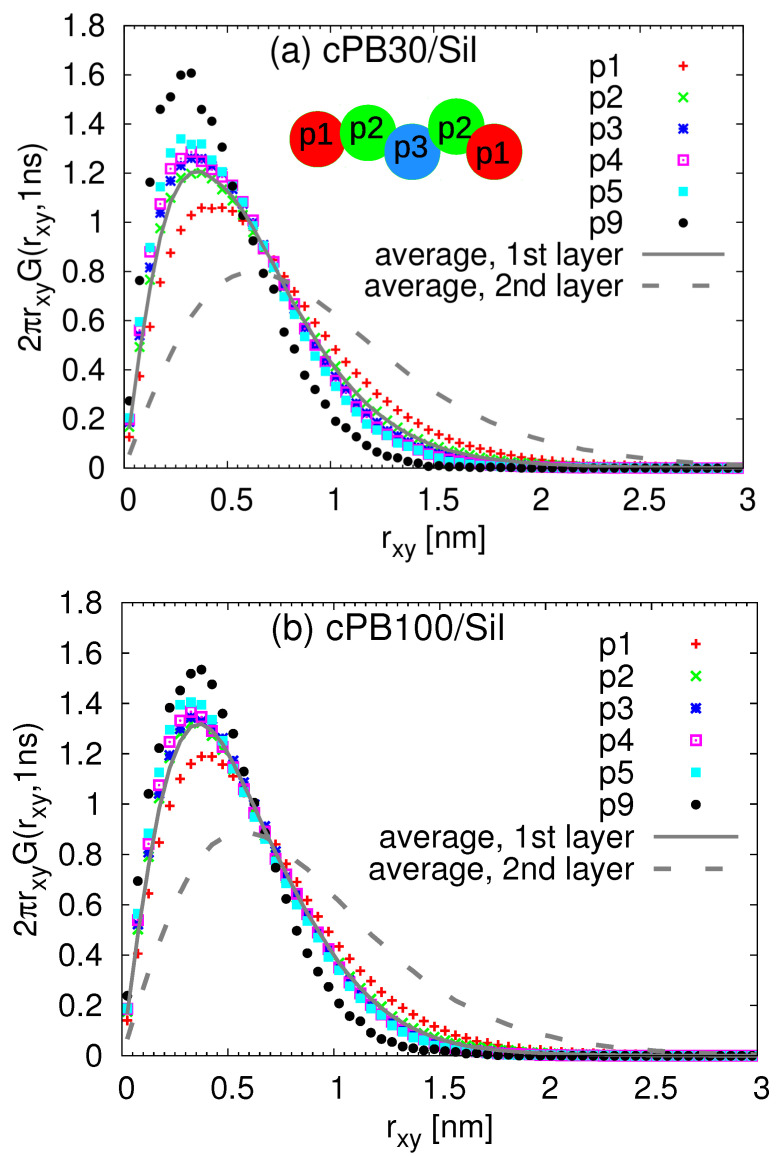
Van Hove functions including lateral displacement of the train monomers for t=1 ns for the (**a**) cPB30/Sil and (**b**) cPB100/Sil system. The legend denotes the position of the monomer in the trains (see the notation in the inset). The lines show the data identical to those presented in Figure 7b and Figure 8b.

Focusing now on the coupling between spatial and dynamical heterogeneities within the train, i.e., on the relation between the position of the monomer within the train and its dynamics, we calculated the lateral displacement for the monomers placed in the train and plotted the Van Hove functions at time t=1 ns for the same positions in the train in Figure 14. More specifically, the labelling of the positions starts from the terminal monomer, i.e., the terminal monomer is labelled as the first one (p1 in Figure 14), the adjacent one as p2 and so on. Considering the symmetric character of the trains and their dynamics observed also in Figure 10, we adjusted the labelling and both terminal monomers are labelled as p1, those connected to them as p2 and so on. Note that in this notation (shown in the inset of Figure 14a for an example of train of five monomers), the number of monomers with different labels is not equal to the length of the train, as it was in the case of data presented in Figure 10. Confronting the data in Figure 14 with those in Figure 13, it seems that the length of the train has a stronger effect on the lateral diffusion of the monomers than the actual position of the monomer in the train.

As a last part of our analysis and to discuss the time scales involved in the heterogeneous dynamics of the adsorbed chains below, we studied the desorption process and computed a characteristic desorption time. We define the adsorption state at the given time *t* from the time origin $t_{0}$ as $S(t+t_{0})=0$ when the monomer is not placed in the adsorption layer (i.e., its distance from the slab is longer than 0.6 nm) and $S(t+t_{0})=1$ when the monomer is adsorbed. Then, we can probe the desorption dynamics by calculating the autocorrelation function of the adsorption status *S* with the following equation [40,54]:

(5) $$C_{\mathrm{ads}}=\langle S\left(t+t_{0}\right)\cdot S\left(t_{0}\right)\rangle .$$

**Figure 15:**
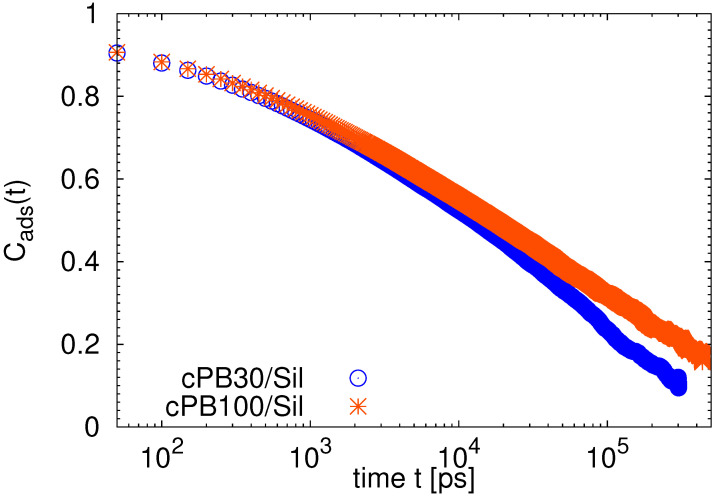
Autocorrelation functions of adsorption status $C_{\mathrm{ads}}$. The error bars are of the order of the symbol size.

Note that this function examines not only the continually adsorbed states (i.e., the situations when the monomer stayed in the adsorbed layer during the whole time window *t*) but also accounts for the events when the monomer was re-adsorbed. In other words, it is non-zero for all cases when the monomer is adsorbed at a reference time (at $t_{0}$) as well as after time *t* (at $t_{0}+t$), independently of its status between these two states. The decorrelation of $C_{\mathrm{ads}}$ is a very slow process, as shown in Figure 15, and the functions decay at time scales of an order of magnitude longer that those found for the longest trains in Figure 11. As cPB100 forms longer and, thus, slower trains than cPB30 (see Figure 5 and Figure 11), it would be in line with a slower relaxation of $C_{\mathrm{ads}}$ of cPB100 in Figure 15. However, as the population of such trains is very low and a characteristic time for the $C_{\mathrm{ads}}$ function (defined at the time when the function decays to 1/e value) is about 24 times longer than the slowest relaxation time found for the long trains in Figure 11, the most plausible explanation for the slow decorrelation of the adsorption states is that the probability of readsorption after a desorption process is high. More specifically, monomers may become a part of the loop (i.e., leaving the nearest vicinity of the substrate) and diffuse back into the adsorption layer after some time *t*, which would be reflected in the $C_{\mathrm{ads}}$ function as a positive $S\left(t+t_{0}\right)\cdot S\left(t_{0}\right)=1$ event. This changes between layers and/or configurations would also be in agreement with the observations in Figure 13. Concerning the differences between the two studied polymer chains, slower desorption dynamics for united atoms of cPB100 with respect to those of cPB30 were also reported for chains adsorbed on silica nanoparticles [54]. In our case the actual decorrelation times are 63 ns for the 100-mer and 36 ns for the 30-mer.

In summary, we showed that the lateral diffusion and the segmental relaxation of the adsorbed monomers are strongly affected by their configurational freedom—more specifically, by the number of their “bonding partners” adsorbed on the surface. Consequently, we speculate that the overall interfacial dynamics in films with high percentage of faster short trains may differ from the films with more populated longer trains, even if the packing near the surface and, thus, the interfacial density would be the same in both systems. Meanwhile, the difference in the interfacial adhesion would also affect the mechanical properties of the nanostructured material.

## 4. Conclusions

We used atomistic molecular dynamics simulations to obtain insights into structure–dynamics relations in thick films of *cis*-polybutadiene (cPB) chains placed between rough amorphous silica slabs in the regime, where the dynamics are not strongly affected by the topological constraints (i.e., entanglements). Particular attention was paid to linking the conformational behavior of cPB chains of two different molecular weights (a unentangled Rouse-like and a mildly entangled one) with their segmental dynamics. Concerning the effect of the position of the polymer segment with respect to the substrate (i.e., spatial effects) on the average structural and dynamical properties, we showed that the lateral translational and orientational dynamics were slowed down near the silica slab and the extension of this effect was in line with the range of the density fluctuations related to the enhanced chain packing near the surface. The degree of the dynamical heterogeneity related to the segmental relaxation, which is quantified by the stretch exponent β, exhibited a non-monotonous dependence on the distance between the polymer and silica slab, most likely due to the altering degree of heterogeneity of the monomer local environment in contrast to a monotonous dependence previously reported for some flat surfaces.

Concerning the dynamics of the particular conformational arrangements (i.e., trains, loops, tails and free segments), spatial effects were also present, and the segments associated with the given conformation relaxed slower when located closer to the silica surface. The segments belonging to loops displayed a slower dynamical response than the tail segments positioned in the same distance from the substrate. Strong internal (positional) dynamical heterogeneities for train segments were detected as a result of the interplay between the entropic and enthalpic interactions with the rough solid substrate. A symmetric dynamical gradient along the trains was observed for characteristic relaxation times, with the slowest components of the film being the internal segments of trains consisting of multiple (more than three) monomers. Consequently, quantitative similarities between the lateral diffusion of the single-monomer trains and of the tail or loop segments in the nearest vicinity of the adsorbed layer were found, indicating dynamical correlations in the film. We expect that the spatio-temporal link between the configurational and dynamical properties presented here will contribute to a better understanding of the experimentally observed interfacial phenomena. In addition, by focusing on the detection and analysis of the slow and fast components in polymer films, we aim to facilitate the description of the coexistence of fast and slow chain populations previously studied by simpler coarse-grained models on heterogeneous surfaces [32,33] and, thus, to go a step further in elucidating dynamic heterogeneities in nanoscopically confined polymers.

## Figures and Tables

**Table 1:** Details about the systems under study. $R_{g}$ denotes the average radius of gyration of the polybutadiene chains, namely $R_{g}={\langle R_{g}^{2}\rangle }^{0.5}$ (*T* = 413 K).

System	Number of Chains	Length of the Chain (Number of Monomers)	$R_{g}$ (nm)	Film Thickness (nm)
cPB30/Sil	261	30	1.434 ± 0.005	13.77
cPB30 bulk	100	30	1.439 ± 0.005	-
cPB100/Sil	90	100	2.70 ± 0.10	15.5
cPB100 bulk	100	100	2.79 ± 0.03	-

## Data Availability

The data that support the findings of this study are available within the article and in Supplementary Materials.

## Supplementary Materials

The following are available online at https://www.mdpi.com/article/10.3390/nano11082075/s1, Description of the fitting procedure, Figures S1, S3 and S4a: Examples of the fitting procedures, Figure S2: The parameters from the KWW function describing long time relaxation of PB100/Sil, Figure S4b: Relaxation times for the segments along the train for trains of different length for cPB100/Sil using different fitting procedure, Figure S5: Average distance from the substrate for the train segments as a function of the position in the train.
