# Supplementary material for: Coupling between Polymer Conformations and Dynamics Near Amorphous Silica Surfaces: A Direct Insight from Atomistic Simulations
Source: Nanomaterials (Basel). 2021 Aug 16;11(8):2075. doi: 10.3390/nano11082075 (PMC8401719; doi:10.3390/nano11082075)
Supplement: Supplementary file 1 [file nanomaterials-11-02075-s001.zip › nanomaterials-1329122-supplementary.pdf]

# SUPPLEMENTARY INFORMATION: Coupling between polymer conformations and dynamics near amorphous silica surfaces: a direct insight from atomistic simulations

Petra Bačová,[1,2] Wei Li,[3] Alireza F. Behbahani,[1] Craig Burkhart,[4]
Patrycja Polińska,[5] Manolis Doxastakis,[3] and Vagelis Harmandaris[1,6,2]

*[1]Institute of Applied and Computational Mathematics (IACM), Foundation for Research and Technology Hellas (FORTH), GR-70013 Heraklion, Crete, Greece*
*[2]Computation-based Science and Technology Research Center, The Cyprus Institute, 20 Constantinou Kavafi Str., Nicosia 2121, Cyprus*
*[3]Department of Chemical and Biomolecular Engineering, University of Tennessee, Knoxville, Tennessee 37996, USA*
*[4]The Goodyear Tire and Rubber Company, Akron, Ohio 44305, United States*
*[5]Goodyear S.A., Colmar-Berg L-7750, Luxembourg*
*[6]Department of Mathematics and Applied Mathematics, University of Crete, GR-70013 Heraklion, Crete, Greece*

*Obtaining the segmental relaxation times*

The Legendre polynomial (correlation function) was calculated for the vectors $\mathbf{v}$ along the backbone connecting the first and the last united atom in the monomer (i.e., 1-4 vector) in the form:

$$C_1(t) = \left\langle \frac{\mathbf{v}(t+t_0)\cdot\mathbf{v}(t_0)}{|\mathbf{v}(t+t_0)||\mathbf{v}(t_0)|} \right\rangle . \tag{1}$$

Average was done over different time origins $t_0$ and over all possible vectors in the given configuration in the given layer. The layer assignment and the classification of the configuration (i.e., train, loop, tail and free) was done at the time $t_0$, it means we probe diffusive behavior and the monomer can rearrange its position during the $t + t_0$ interval. In the case of the 30-mer PB, we fit each correlation function for a given layer and for a given configuration (i.e., train, loop, tail and free) by a Kohlrausch-Williams-Watts (KWW) stretch exponential function:

$$f(t) = \exp\left(-(t/\tau_\mathrm{i})^{\beta_i}\right) \tag{2}$$

where $\beta$ is a stretch exponent and $\tau_\mathrm{i}$ is a characteristic relaxation time with "i" being a label denoting "tn" (train), "l" (loop), "tl" (tail) and "f" (free) segments. For the slightly entangled PB100, we used a sum of two KWW functions to capture the slow relaxation modes after the primary, segmental relaxation:

$$f(t) = A\exp\left(-(t/\tau_\mathrm{i}^{'})^{\beta_\mathrm{i}^{'}}\right) + (1-A)\exp\left(-(t/\tau_\mathrm{i}^{''})^{\beta_\mathrm{i}^{''}}\right) \tag{3}$$

where $A$ is a prefactor smaller than 1. In the following we use the single apostrophe for the primary relaxation (i.e., the main decay of $C_1(t)$). Examples of the fitting procedure for the two studied systems are shown in Fig. S1 for the vectors $\mathbf{v}$ averaged over all monomers in the given layer ($d$ in Fig. S1 denotes the distance of the given layer from the substrate), i.e., for the case where no distinction of the train, loop, tail and free configuration was made. The bad separation of the slow and fast relaxations in cPB100/Sil systems makes the fitting procedure very challenging and led to bigger error bars in the reported stretch exponents for this system (compare Fig.10(c) and (d) in the main manuscript). This issue is also the reason why the stretch exponents of the second, slow relaxation in the case of trains, $\beta_\mathrm{tn}^{''}$, are so high (see Fig. S2(b)). The smooth decay of $C_1(t)$ in the case of train segments is followed by a sudden drop (see the red points in Fig. S1(b)), which can be approximated by an exponential function with the stretch exponent of 1, i.e., by the Debye relaxation function. Aware of this phenomenon, we set up $\beta_\mathrm{tn}^{''}$ equal to 1 when performing the fitting procedure for the correlation functions corresponding to trains of different length in the case of cPB100/Sil system. An example of the fitting procedure for the train consisting of 7 monomers is shown in Fig. S3(a) for the cPB30/Sil system and in Fig. S3(b) for the cPB100/Sil system.

As the sudden drop in $C_1(t)$ corresponding to the train segments of 100-mer PB occurs at $C_1(t) < 0.1$, another approach to describe the main decay would be to match the data to a single stretch exponential function, i.e., to eq. 2. The results and an example of the fitting procedure for such an approach are presented in Fig. S4. We report this approach to complete the picture and to demonstrate that both approaches (i.e., using either eq. 2 or eq. 3) give consistent results. We note that the normalized relaxation times shown in Fig. S4(b) are in agreement with the data presented in Fig. 11(b) of the main manuscript and the majority match within the error bars.

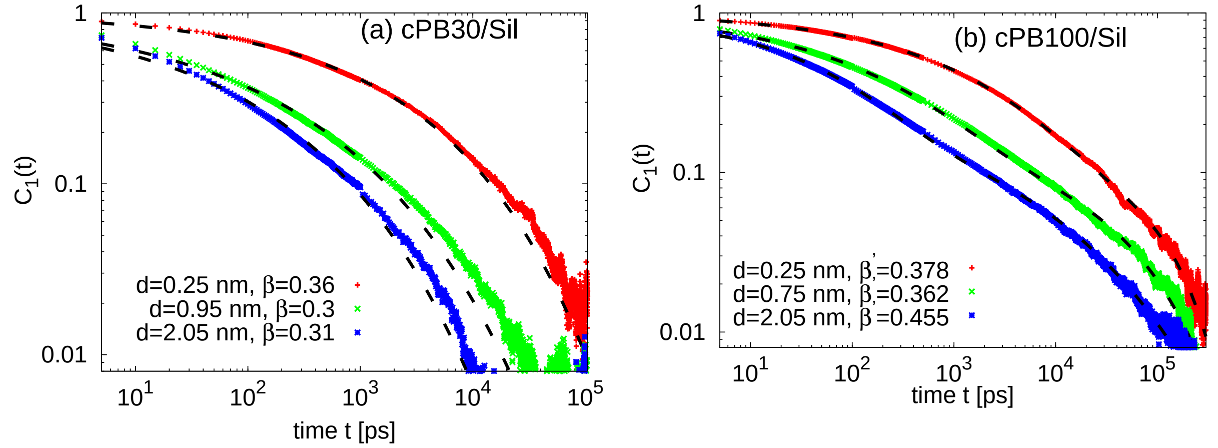


FIG. S1: Examples of the fitting procedure for (a) cPB30/Sil using eq. 2 and (b) cPB100/Sil system using eq. 3. The simulation data are plotted with points, lines represent the fitting functions. The correlation functions were averaged over all monomers in the given layer; the distance of the layer from the substrate $d$ can be found in the legend together with the stretch exponent $\beta$ for the particular fitting function.

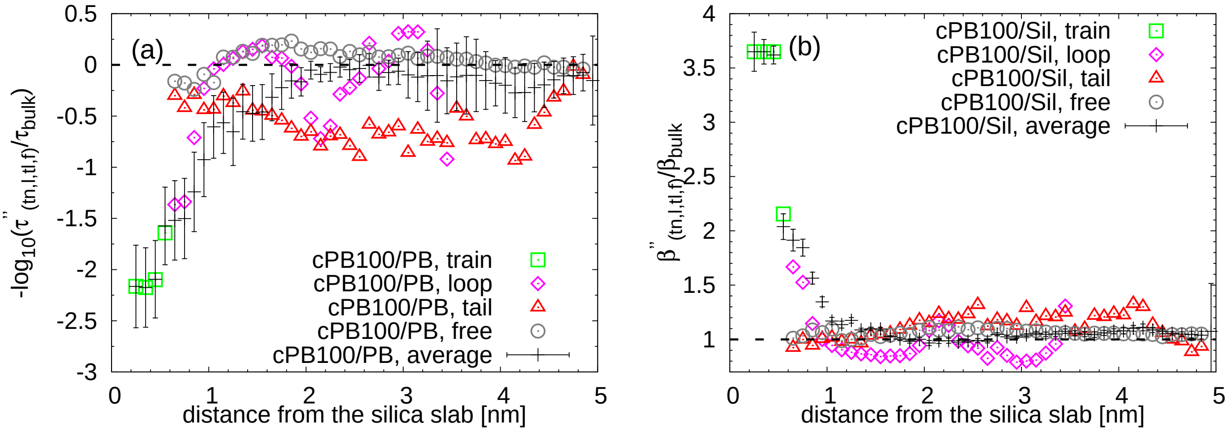


FIG. S2: The parameters from the KWW function describing long time relaxation ($\tau''$, $\beta''$) for cPB100/Sil normalized by the corresponding bulk values.

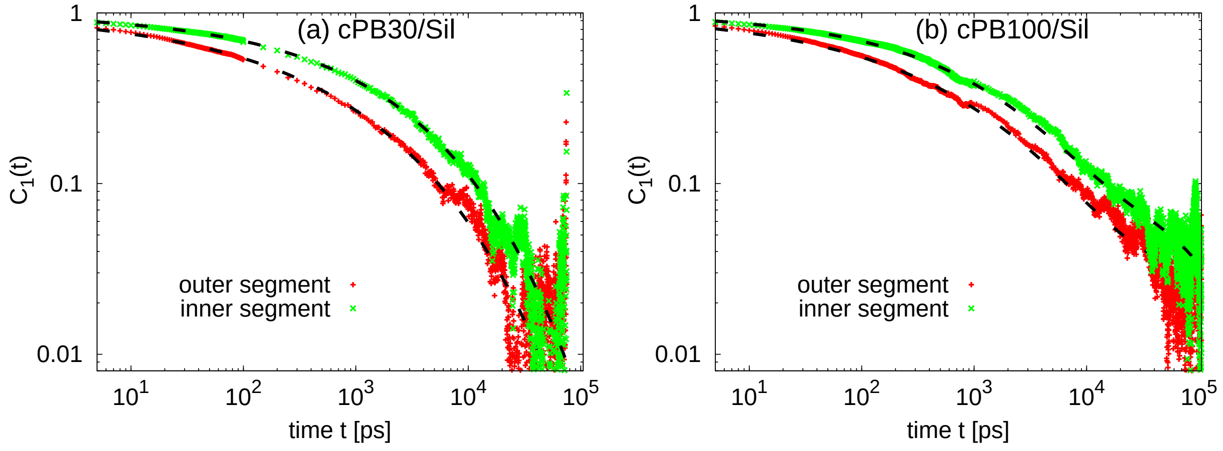


FIG. S3: Examples of the fitting procedure for (a) cPB30/Sil using eq. 2 and (b) cPB100/Sil system using eq. 3. The simulation data are plotted with points, lines represent the fitting functions. The correlation functions were calculated for trains of length of 7 monomers and the label "outer segment" corresponds to the terminal segment (i.e., the first monomer of the train) and the label "inner segment" corresponds to the monomer in the middle of the train (i.e., the fourth monomer of the train).

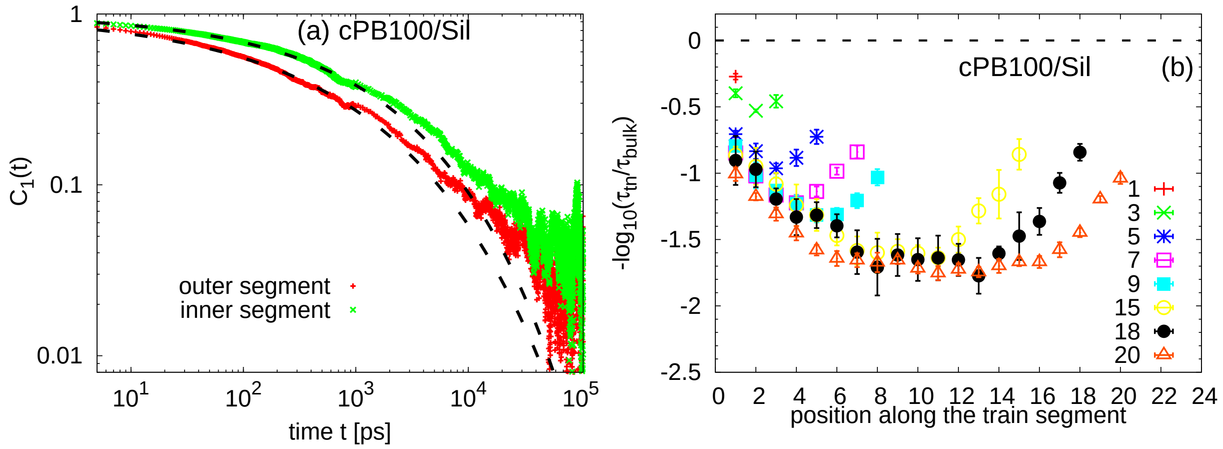


FIG. S4: Examples of the fitting procedure and results for cPB100/Sil system using eq. 2. In (a) the simulation data are plotted with points, lines represent the fitting function. The correlation functions were calculated for trains of length of 7 monomers and the label "outer segment" corresponds to the terminal segment (i.e., the first monomer of the train) and the label "inner segment" corresponds to the monomer in the middle of the train (i.e., the fourth monomer of the train). In (b) the results are normalized by the corresponding bulk value.

*Relation between the distance from the surface and position in the train configuration*

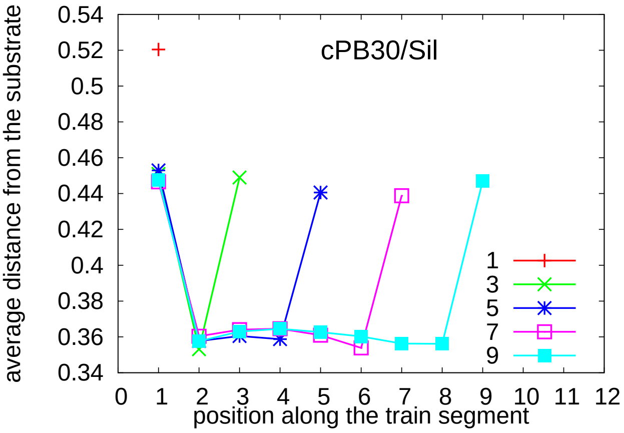


FIG. S5: Average distance from the substrate (in nm) for the train segments as a function of the position in the train for cPB30/Sil.
